# Magnetic resonance imaging incidents are severely underreported: a finding in a multicentre interview survey

**DOI:** 10.1007/s00330-021-08160-w

**Published:** 2021-07-20

**Authors:** Johan Kihlberg, Boel Hansson, Annika Hall, Anders Tisell, Peter Lundberg

**Affiliations:** 1 Department of Radiology in Linköping and Department of Health, Medicine and Caring Sciences, Linköping University, Linköping, Sweden; 2 Center for Medical Image Science and Visualization (CMIV), Linköping University, Linköping, Sweden; 3 Department of Medical Imaging and Physiology and Department of Diagnostic Radiology, Clinical Sciences, Lund University, Lund, Sweden; 4 Department of Radiation Physics and Department of Health, Medicine and Caring Sciences, Linköping University, Linköping, Sweden

**Keywords:** Medical device safety, Magnetic resonance imaging, Incident reporting, Patient safety

## Abstract

**Objectives:**

The purpose of this study was to develop a procedure to investigate the occurrence, character and causes of magnetic resonance (MR) imaging incidents.

**Methods:**

A semi-structured questionnaire was developed containing details such as safety zones, examination complexity, staff MR knowledge, staff categories, and implementation of EU directive 2013/35. We focused on formally reported incidents that had occurred during 2014–2019, and unreported incidents during one year. Thirteen clinical MR units were visited, and the managing radiographer was interviewed. Open questions were analysed using conventionally adopted content analysis.

**Results:**

Thirty-seven written reports for 5 years and an additional 12 oral reports for 1 year were analysed. Only 38% of the incidents were reported formally. Some of these incidents were catastrophic. Negative correlations were observed between the number of annual incidents (per scanner) and staff MR knowledge (Spearman’s rho − 0.41, *p* < 0.05) as well as the number of MR physicists per scanner (− 0.48, *p* < 0.05). It was notable that only half of the sites had implemented the EU directive. Quotes like ‘*Burns are to be expected in MR*’ and not even knowing the name of the incident reporting system suggested an inadequate safety culture. Finally, there was a desire among staff for MR safety education.

**Conclusions:**

MR-related incidents were greatly underreported, and some incidents could have had catastrophic outcomes. There is a great desire among radiographers to enhance the safety culture, but to achieve this, much more accessible education is required, as well as focused involvement of the management of the operations.

**Key Points:**

*• Only one in three magnetic resonance–related incidents were reported.*

*• Several magnetic resonance incidents could have led to catastrophic consequences.*

*• Much increased knowledge about magnetic resonance safety is needed by radiologists and radiographers.*

**Supplementary Information:**

The online version contains supplementary material available at 10.1007/s00330-021-08160-w.

## Introduction

Patient safety is an important aspect of health care, and the risk of injury in health care is much greater than the risk of being injured during a commercial flight [1]. Systematic preventive work is required and everyone in Swedish health care is required by regulations to carry out this type of work [2]. Great resources are spent on identifying and preventing infections, which is good and affects everyone in the health care sector [3]. But there are areas that have not yet been mapped, such as the security of magnetic resonance (MR) imaging. There are reports of accidents involving serious injuries and even death [4].

**Fig. 1:**
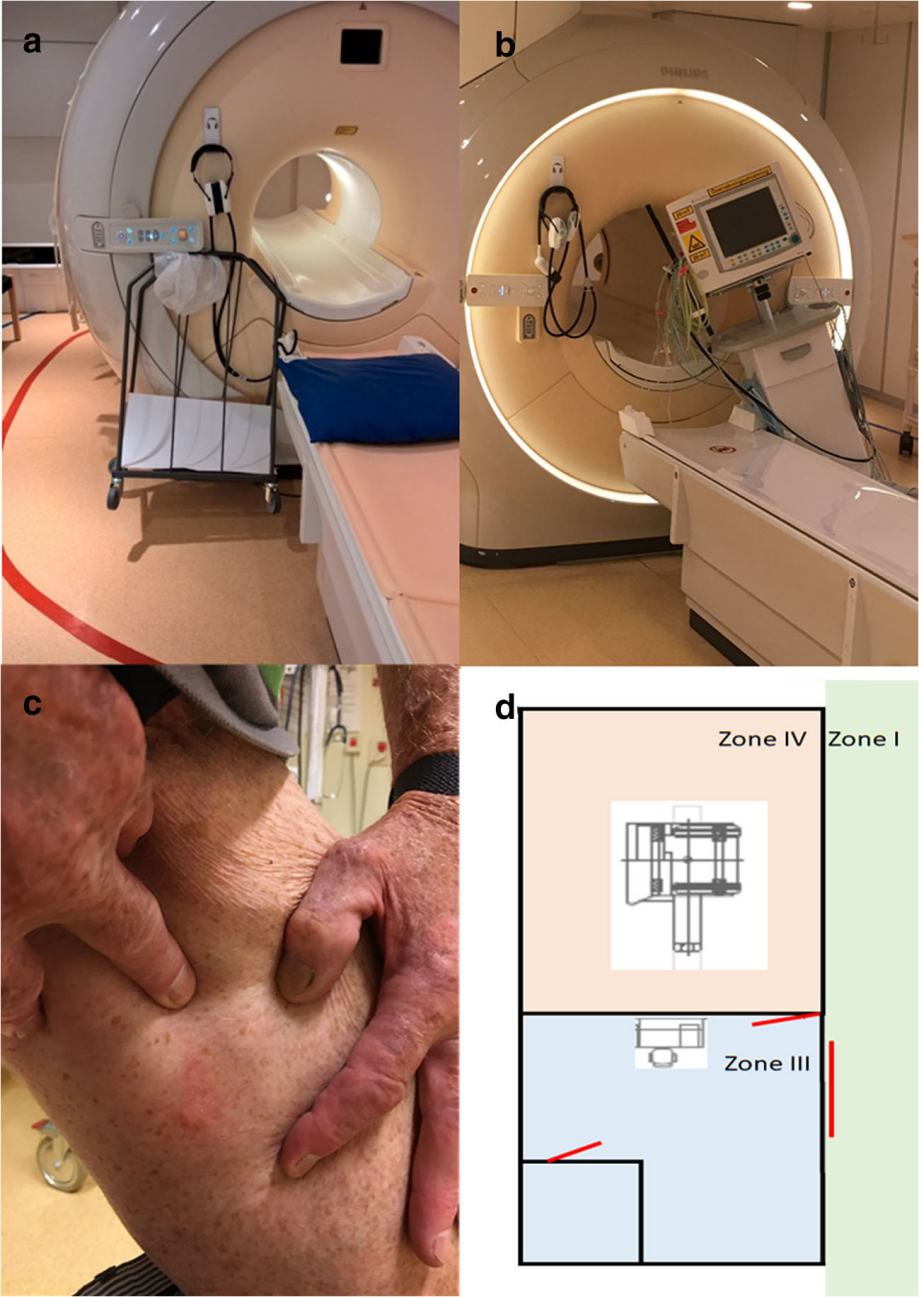
Examples of MR accidents. In the top left corner (**a**), a trolley is stuck on the magnet. In the top right corner (**b**), a patient monitor is stuck on the magnet. In the lower left corner (**c**), a patient suffered a blister in the groin. In the lower right corner (**d**), safety zone II is missing (the red lines represent the three doors to the unit)

MR is associated with two different major risks, static and time-variant fields. Firstly, since the static magnetic field (or B0) is always on, there is always a risk that if objects containing ferromagnetic metals come close to the magnet, they will turn into projectiles that are pulled into the magnet; see Fig. 1a, b for two such recent examples. Secondly, MR is performed using two types of time-varying spatially oriented electromagnetic fields, the radio frequency (RF) field (or B1 field; specifically, the RF transmission field; B1 + rms) and the gradient field. A primary risk associated with the static magnetic field is presented by objects that are ferromagnetic and can act as dangerous projectiles, or with a torque of ferromagnetic implants in human subjects. The primary risk of RF is associated with electrically conductive objects (magnetic or non-magnetic, or electrically conductive, including carbon fibres, electrolytes and even healthy tissues) where burns can occur. Skin-to-skin contact on the patient can also lead to severe burns, as shown in Fig. 1c. General discomfort caused by the application of RF power including heat sensations and a general temperature rise in the patient can also occur, and this is something that can be particularly disturbing in certain patient groups, including geriatric or diabetic patients. Finally, there are risks associated with the time-varying magnetic field gradients which may induce electrical currents in the body, which can damage implants containing electronics and cause discomfort in the form of peripheral nerve stimulation (PNS). These varying magnetic fields also cause a very high acoustic noise level that is associated with an MR examination, and this can lead to hearing loss, tinnitus and discomfort [5]. There are also other risks that appear in the context of MR examinations, such as injection of contrast agents (containing gadolinium ions) [6], but the pharmacological aspects will not be addressed here.

To minimise the risks to patients, there are international guidelines on risk management, in which the magnetic field is limited to 8 tesla (T) for clinical systems, the amount of RF power is limited to e.g. 4 W/kg (whole body weight), and the varying magnetic field gradients (e.g. slew rate and amplitude) are limited to reduce potential harmful effects or discomfort [7]. Additionally, there is also a set of European Union directives subsequently implemented in national laws, which regulate the working environment for employees who work in MR environments [8]. Implants will increase the risk for harmful consequences, and appropriate testing of implants should (ideally) be performed by all manufacturers of medical devices. Such documentation must be available to MR staff prior to an examination. However, such testing is neither universally compulsory nor fully standardised (and sometimes not even understood), even though clear guidelines are available [9]. Based on such MR tests of implants, certain parameters in the MR scanner can be limited (as advised by the provided MR conditions) to reduce the risk of patient injury. An important aspect is the safety rules that are imposed at the planning stage in the MR unit. The recommendations on floor planning include implementing four zones, ranging from zone I, where the public has free access, to the restricted (zone II) and locked zones III and IV, the latter being the room with the MR magnet [10]. Figure 1D shows a floor plan for an actual MR unit where zone II is missing; MR safety was not prioritised at the stage of planning this unit.

Yet another aspect is the staff’s depth of knowledge about MR safety. Westbrook and Talbot measured radiographers’ knowledge about MR, and they found that MR safety knowledge in particular was very low [11]. Hudson et al also pointed out that the low level of MR safety knowledge of radiographers could cause incidents [12].

**Fig. 2:**
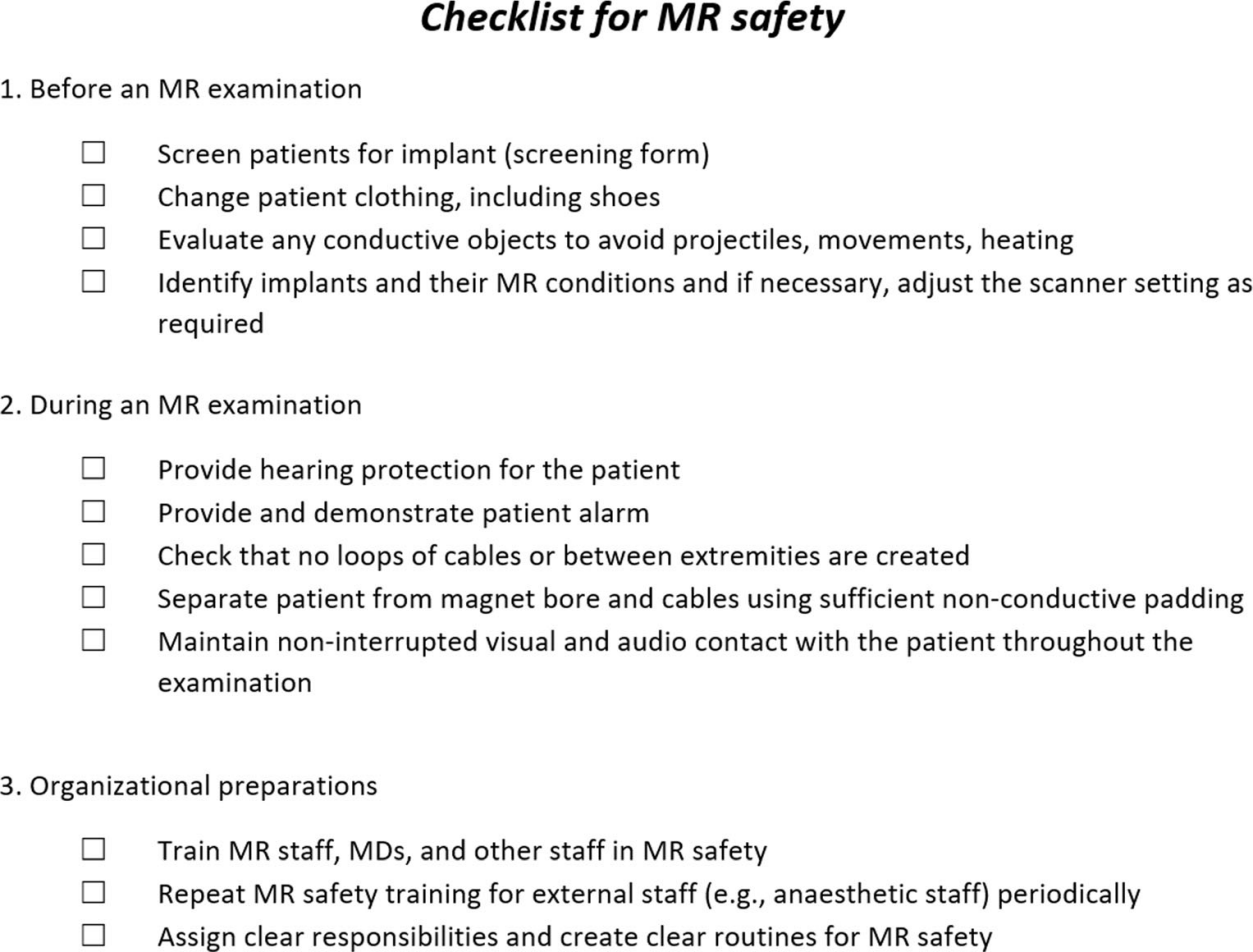
Checklist for MR safety. A checklist lightly based on The Society for MR Radiographers & Technologists’ safety poster [13]

Checklists can be used to cover a range of MRI safety aspects (see Fig. 2 for an overview of the most important aspects, which is based on a contribution to the Society for MR Radiographers & Technologists (SMRT)) [13]. Briefly, there are three steps at the scanner: before, during, and after scanning. All patients need to (i) be screened before the scanning using a written screening form, (ii) be prepared for an examination by changing clothing (flame resistant) and (iii) adapt to the scanning protocol according to the specific conditions of the patient and any potential implants. During the examination, hearing protection is needed, cables or extremities must not form conductive loops, the patient should also not touch the bore of the magnet and communication should be established. Beyond these basic steps, the operation also requires a mandatory and well-developed safety structure at the organisational level, such as developed routines for a low threshold incident reporting system and continuous education of staff in new advances in MR safety as well as clear responsibilities between staff categories [13, 14]. The Swedish Patient Safety Act (SFS 2010:659) dictates that all incidents must be reported to the appropriate authorities and an investigation should be made to help avoid such events in the future. An incident could be an event where someone is injured, but also when a situation arises that could potentially lead to an injury to staff, a patient, or a family member, or damage to equipment [2]. Incidents appear to be greatly underreported [15]. In Denmark, a national incident database of MR accidents and incidents exists, but a recent study reported a serious level of missing reports also in that database [16].

In order to increase the safety of MR examinations, and to reduce the incidence of future accidents, we clearly must learn about what kinds of MR-related incidents occur in clinical routines, and the possible reasons why they occur. The purpose of this study was (i) to develop a site-focused qualitative and quantitative model for investigating the rate and severity of MR-related incidents and its circumstances, and (ii) to investigate the nature of MR incidents that occurred during a 5-year period. The target group being studied was all health care facilities that have MR scanners in the Swedish South East Health Care Region.

## Materials and methods

A semi-structured questionnaire, partly based on questions from a previous national survey [17], was developed (see supplement). It included questions about background conditions such as the site floor zones of the MR unit, the level of complexity of MR examinations, which categories of staff worked at the MR unit, the responsibilities of each profession, how incidents were handled, how the implementation of EU directive 2013/35 (Swedish law AFS 2016:3) was dealt with, the level and extent of MR training, fundamental MR safety knowledge among staff and the level of collaboration with other units on MR safety. We also acquired a list of documented incident reports from the previous 5 years (June 2014–June 2019) at the site, and an oral description of additional undocumented incidents during the last year (June 2018–May 2019). In addition, we enquired about the staff’s thoughts on how to improve the MR safety level at the site. The questions are described in more detail in the supplement. The documentation of the survey was later reviewed, analysed and classified by an expert panel (J.K., A.H., B.H.).

All MR units in the Swedish South East Health Care Region (with a total population of 1.1 million inhabitants), both public and commercial, were contacted to request participation. The region has mainly public hospitals, but also a few commercial health care enterprises with MR scanners. There were five local hospitals, three regional hospitals, one university hospital and two commercial MR units in the study. There are a total of 20 MR scanners, which were clustered into 13 separate sites. The responsible radiographers (respondents) at each MR site were interviewed, and the questionnaire was provided 4 weeks before the site visit. As no patient-sensitive information was collected, approval by the national ethics committee was not required. Nevertheless, written information on the study and assurance that participation was voluntary and confidential were given in advance to staff at each site.

Our evaluation of the floor zones was based on recommendations [18] and scored by the number of safety zones in each MR unit. ‘Complexity of MR examinations’ was assessed by how many different types of examinations were performed in the unit, with scores ranging from 1 to 8. ‘Level of MR knowledge’ was judged by requesting the responsible radiographer on the site to report the fraction of staff who would be able to adjust five relevant safety parameters at the MR scanner console [7, 14]; the numbers were then reduced to the mean of the five parameters.

Notes from the interview were filed the same day. Open questions were analysed by conventional content analysis [19]. An incident was defined as an event that caused, or that could have caused, injury or damage [2]. Both reported incidents, and undocumented events, were classified into degree of severity (with respect to both worst-case possible scenario and actual outcome) as well as risk for repetition.

Severity was subdivided into four levels: (1) minor—discomfort or insignificant injury, (2) moderate—short-term disability, (3) significant—persistent moderate disability and (4) catastrophic—death or major disability. The risk of recurrence was graded as follows: (1) very small—could occur once a year or less, (2) small—could occur each month, (3) large—could occur each week and (4) very large—could occur daily [20]. The grading was done by three experienced MR radiographers (J.K., A.H., B.H.), who discussed until consensus was reached. The radiographers were blinded to the studied institutes, and each had more than 10 years of experience of MR safety.

The incidents were also categorised into (1) thermal, (2) mechanical, (3) projectile, (4) peripheral nerve stimulation and (5) miscellaneous. This categorisation was adopted from a study by Delfino et al [15]. The ‘actual reporting rate’ (*R*) was calculated in the following manner:

$$ R={n}_{WR}/\left({n}_{WR}+{n}_{DI}\right),\left[ 0\le R< 1\right], $$

where *n*_*WR*_ is the ‘number of in writing documented incident reports’ and *n*_*DI*_ is the ‘undocumented orally described incident reports’.

The parameters from the questionnaire were at nominal or ordinal levels, and nonparametric tests (Mann-Whitney U Test, chi^2^ and Spearman correlation) were performed. SPSS (IBM Inc) was used for the statistical treatment, and the significance level was set at *p* < 0.05.

## Results

### Site-focused model

All approached respondents chose to participate, i.e. 13 sites with a total of 20 scanners with a field strength of 0.2–3 T. Thirty-eight documented incidents occurred between June 2014 and May 2019, and 24 incidents reported orally but undocumented in writing occurred between June 2018 and May 2019, at the 13 sites. Two respondents, with three scanners, were unable to even search for MR-related incidents 5 years back in their incident report system, as their incident reporting system did not allow searches for MR-related incidents. The respondents at another two sites did not know the name of their local incident reporting system. However, all respondents knew how to fill in an incident report at their site, and it was claimed that all the personnel did as well. Four respondents claimed that not a single MR incident had occurred during the last 5 years, at their site.

### Incidents and background factors

Table 1 shows an overview of the sites and the number of incidents, number of scanners, examinations per year, staffing at the scanner, self-estimated MR knowledge, whether anaesthesia or external cleaners are used, how complex the examinations are, how many MR physicists affiliated and number of floor safety zones.

**Table 1:** Overview of incidents and background factors

Site	Number of scanners	Written incidents 2014–2019	Oral incidents 2019	Incidents/scanner/year^a^	Examinations/scanner 2018^b^	Minimum staff/scanner^c^	MR knowledge^d^	Anaesthesia	External cleaners	Examination complexity^e^	Physicist/ scanner^f^	Number of zones^g^
Site 1	1	1	0	0.2	600	1.0	25%	–	X	1	0.00	3
Site 2	2	2	0	0.2	1727	1.5	100%	X	X	7	0.45	4
Site 3	2	11	1	1.6	1823	1.5	50%	X	X	2	0.45	4
Site 4	2	13	1	1.8	2270	1.5	100%	X	X	6	0.45	3
Site 5	2	0	3	1.5	2814	1.5	20%	–	–	2	0.45	4
Site 6	1	6	2	3.2	2992	2.0	20%	X	–	4	0.25	3
Site 7	2	0	1	0.5	3249	2.0	67%	X	–	3	0.45	4
Site 8	1	1	2	2.2	3779	2.0	50%	–	–	2	0.25	4
Site 9	2	N/A	2	1.0	3812	2.0	20%	X	X	5	0.25	4
Site 10	2	3	1	0.8	4307	1.0	40%	X	X	6	0.25	4
Site 11	1	0	1	1.0	4500	2.0	20%	–	–	2	0.25	4
Site 12	1	0	0	0.0	4500	1.0	60%	–	X	2	0.08	2
Site 13	1	N/A	8	8.0	4846	2.0	0%	X	–	3	0.25	4
Total/average	20	37	22	1.7	41217	1.6	44%	8	7	3	0.30	3.6

The responding sites are listed with background factors. Columns are described as footnotes below

*N/A* not available

^a^Written incident reports, from 1 June 2014 to 1 June 2019, and additional orally described incident reports, 1 June 2018 to 1 June 2019, were summed up and divided into year and number of scanners at the site

^b^The number of examinations per scanner for the year 2018 and for each site was listed. Note that site 13 just scanned part time

^c^Minimum of staff per scanner were listed, not only radiographers but also staff working close to the scanner

^d^The percentage for ‘MR safety knowledge’ showed the percentage of the radiographers at the site that had MR knowledge of adjusting examinations at the MR scanner based on limitations in ‘Specific Absorption Rate’ (SAR), RF transmission field (B1 + rms), varying gradients dB/dt, ‘Specific Energy Dose’ (SED) and who were able to determine differences in field strength and radio frequency irradiated area relative to an implant, estimated by the radiographer in charge

^e^Sites with anaesthesia and external cleaners are marked. Examination complexity was scored as 1 point for each examination of (a) head, spine and extremities; (b) abdominals; (c) angiography; (d) heart; (e) spectroscopy; (f) fMRIs; (g) anaesthetised patients; and (i) patients with pacemakers

^f^The number of MR physicists was divided by the number of scanners they were responsible for

^g^The number of zones was counted from zone I where the public has free access to zone IV inside the room with the scanner

The total number of incident reports documented in writing during a period of 5 years at 11 sites with 17 scanners (two sites with three scanners could not search for incidents) was 37, which corresponds to 7.4 reports per year, 0.4 reports/scanner/year. In addition, these sites also reported orally 12 additional incidents during the previous year. Consequently, the average proportion of written reports compared to all incidents that occurred corresponded to an actual reporting rate of 38%. Moreover, the number of incidents per year and scanner was 1.1, and of these, only 0.4 was documented in writing. Interestingly, the sites which used their own dedicated cleaning staff to clean the scanner room had more incidents than those using supervised external cleaners (median 1.3 and 1.0, respectively, *p* < 0.05, using Mann-Whitney U test). There was a difference between the number of incidents that involved external staff members (e.g. anaesthesia teams, n = 14) and those that did not (n = 48) (*p* < 0.05, one-sample chi^2^).

**Fig. 3:**
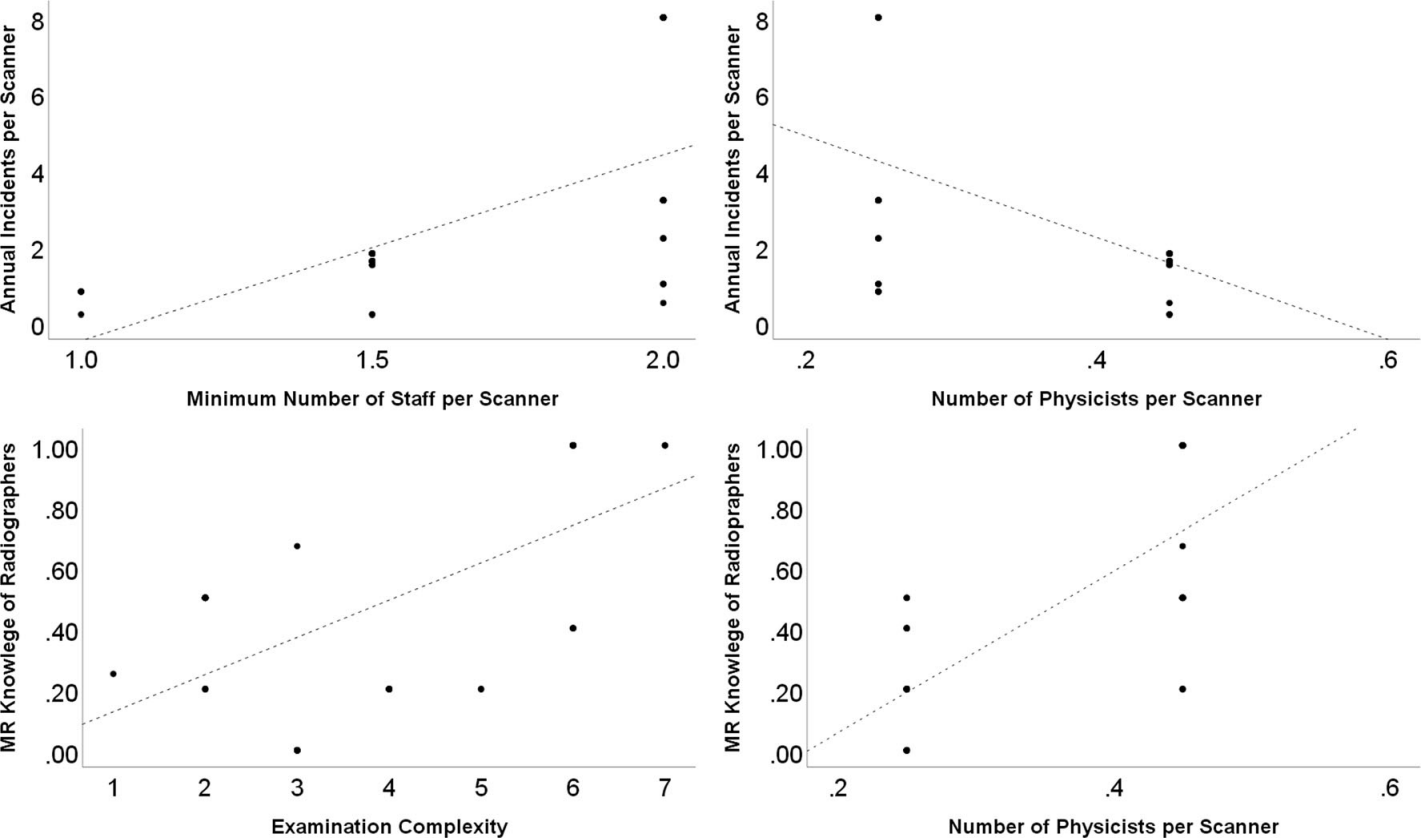
Correlation graphs of some background factors for the incidents and MR safety knowledge of radiographers. Correlation between annual incidents per scanner with staff working with the scanner and staffing of physicists in the top row. In the bottom row, correlation between estimate of radiographer’s MR knowledge and examination complexity and staffing of MR physicists

Figure 3 shows the data in more detail, specifically the correlations between different factors associated with incidents. There was a significant (*p* < 0.05) correlation between (i) incidents per scanner and year and number of staff (MR operators, or MR patient–handling staff) per scanner (Spearman’s rho 0.69), (ii) incidents per scanner and number of MR physicists per scanner (− 0.48) and (iii) level of MR knowledge and examination complexity (0.38), as well as with (iv) the level of MR knowledge and the number of MR physicists per scanner (0.78). Additionally, there was also a correlation between level of MR knowledge (− 0.41) and the number of examinations per year (0.52).

**Fig. 4:**
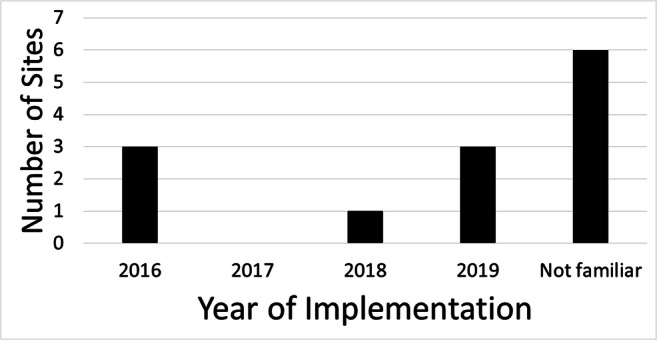
Implementation of EU directive. The responders answered the question concerning which year the Swedish law AFS 2016:3 (implementation of the EU directive 2013/35) was implemented at the site

In Table 2, the 62 incidents are categorised into thermal, mechanical, projectile, PNS and miscellaneous events. The major categories (projectile and miscellaneous events) are respectively divided into subcategories in Tables 3 and 4. At one site, one radiographer stated that ‘*Burns can be expected in MR*’, suggesting that such injuries do not require any formal incident reports. Another interesting fact was that almost half of the responders were unaware of the EU directive 2013/35 (which has been turned into the occupational health law AFS2016:3 in Sweden); see Fig. 4.

**Table 2:** All incidents in categories

Category	Number	Fraction (%)	Consequence severity median	Potential severity median	Probability of recurrence median	Risk assessment median
Peripheral nerve stimulation^a^	1	1.6	2.0	2.0	1.0	2.0
Mechanical^b^	3	4.8	2.0	2.5	1.0	2.5
Thermal^c^	4	6.5	1.0	3.0	2.0	6.0
Projectile^d^	27	43.5	1.0	3.0	1.0	3.0
Miscellaneous^e^	27	43.5	1.0	3.0	1.0	3.0
Total/median	62	100	1.0	3.0	1.0	3.0

Written reported incidents (1 June 2014 to 1 June 2019) and additional orally reported incidents (1 June 2018 to 1 June 2019) categorised and graded for actual consequence severity, potential severity, probability of recurrence and risk assessment. Examples of each category are given as footnotes. The severity grades were (1) minor—discomfort or insignificant injury, (2) moderate—transient disability, (3) significant—persistent moderate disability and (4) catastrophic—death or major disability. The probability of recurrence was graded as (1) very small—could happen once/year or less, (2) small—could occur every month, (3) large—could occur every week and (4) very large—could occur daily. Risk assessment was defined as the product of potential severity and probability of recurrence

^a^One case with unclear pain during imaging

^b^One case of a finger squeezed between the patient table and patient trolley, one obese patient was stuck in the tunnel and one anaesthesia patient had redness after coil pressure

^c^Three cases of redness due to a tattoo, being too close to the coil and wearing synthetic fabric. One case of blisters in the groin due to bilateral hip and knee prostheses

^d^Three cases of large/heavy metal objects, 15 cases of small, blunt metal objects and nine cases of sharp metal objects; for details, see Table 3

^e^Six cases regarding pacemakers or cochlea implants, ten cases regarding other implants, four cases regarding external devices and five cases regarding scanner malfunction; for details, see Table 4

**Table 3:** The projectile incidents in subcategories

Subcategory of projectiles	Number	Fraction (%)	Consequence severity median	Worst case severity median	Probability of recurrence median	Risk assessment median
Large/heavy metal^a^	3	11.1	2.0	4.0	2.0	8.0
Sharp small/medium-size metal^c^	9	33.3	1.0	3.0	2.0	6.0
Blunt small metal^b^	15	55.6	1.5	2.0	1.0	2.0
Total/median	27	100	1.0	3.0	2.0	6.0

Written reported incidents (1 June 2014 to 1 June 2019) and additional orally reported incidents (1 June 2018 to 1 June 2019) subcategorised and graded for actual consequence severity, potential severity, probability of recurrence and risk assessment. Examples of each subcategory are given as footnotes. The severity grades were (1) minor—discomfort or insignificant injury, (2) moderate—transient disability, (3) significant—persistent moderate disability, and (4) catastrophic—death or major disability. The probability of recurrence was graded to (1) very small—could happen once/year or less, (2) small—could occur every month, (3) large—could occur every week and (4) very large—could occur daily. Risk assessment was defined as the product of potential severity and probability of recurrence

^a^Two cases of a physiological monitor on wheels and one case of a roller table which were stuck in the gantry

^b^Two cases regarding pens, one regarding a hairpin, two cases regarding paper clips, two cases regarding coins, two cases regarding telephones, two cases regarding keys and one case each regarding a belt, a stethoscope, a metal name tag, and a metal medicine jar being inside or close to the tunnel

^c^Three cases of wheelchairs, two walkers, one bed, one metal prosthesis and two pairs of scissors being inside or close to the tunnel

**Table 4:** The miscellaneous incidents in subcategories

Subcategory of miscellaneous	Number	Fraction (%)	Consequence severity median	Worst case severity median	Probability of recurrence median	Risk assessment median
Scanner malfunction^a^	3	10.7	1.0	3.0	1.0	3.0
Unauthorised person^b^	3	10.7	1.0	3.0	1.0	3.0
External devices^c^	4	14.3	–	2.5	1.0	2.5
Pacemakers and cochlea implants^d^	6	21.4	2.0	3.0	1.0	3.0
Other implants^e^	11	39.3	1.0	3.0	1.0	3.0
Total/median	27	100	1.0	3.0	1.0	3.0

Written reported incidents (1 June 2014 to 1 June 2019) and additional orally reported incidents (1 June 2018 to 1 June 2019) subcategorised and graded for actual consequence severity, potential severity, probability of recurrence and risk assessment. Examples of each subcategory are given as footnotes. The severity grades were (1) minor—discomfort or insignificant injury, (2) moderate—transient disability, (3) significant—persistent moderate disability and (4) catastrophic—death or major disability. The probability of recurrence was graded to (1) very small—could happen once/year or less, (2) small—could occur every month, (3) large—could occur every week and (4) very large—could occur daily. Risk assessment was defined as the product of potential severity and probability of recurrence

^a^One patient alarm in the scanner was broken when patient vomited, one aborted examination due to spontaneous quench and aborted examination due to power failure

^b^One relative of a patient and one external person entered the scanner room unprepared, and one interpreter failed to translate properly

^c^Three patients with a hearing aid, personal alarm and foot shackle respectively were stopped close to the room and one patient with a foot shackle was examined

^d^Three patients with pacemakers were stopped close to the scanner room, one man with a pacemaker entered the scanner room and two patients with cochlea implants which were dislocated in the scanner

^e^Three patients with splinters of metal were stopped close to the scanner room, one patient with a splinter was examined, one patient with a urine pump was examined, one patient with a loop recorder was examined, one patient with a breast expander was examined, one patient with a glucose meter was stopped close to the scanner room, two patients with brain ventricle shunts were stopped close to the scanner room and one patient with a vagus nerve stimulator was stopped close to the scanner room

**Fig. 5:**
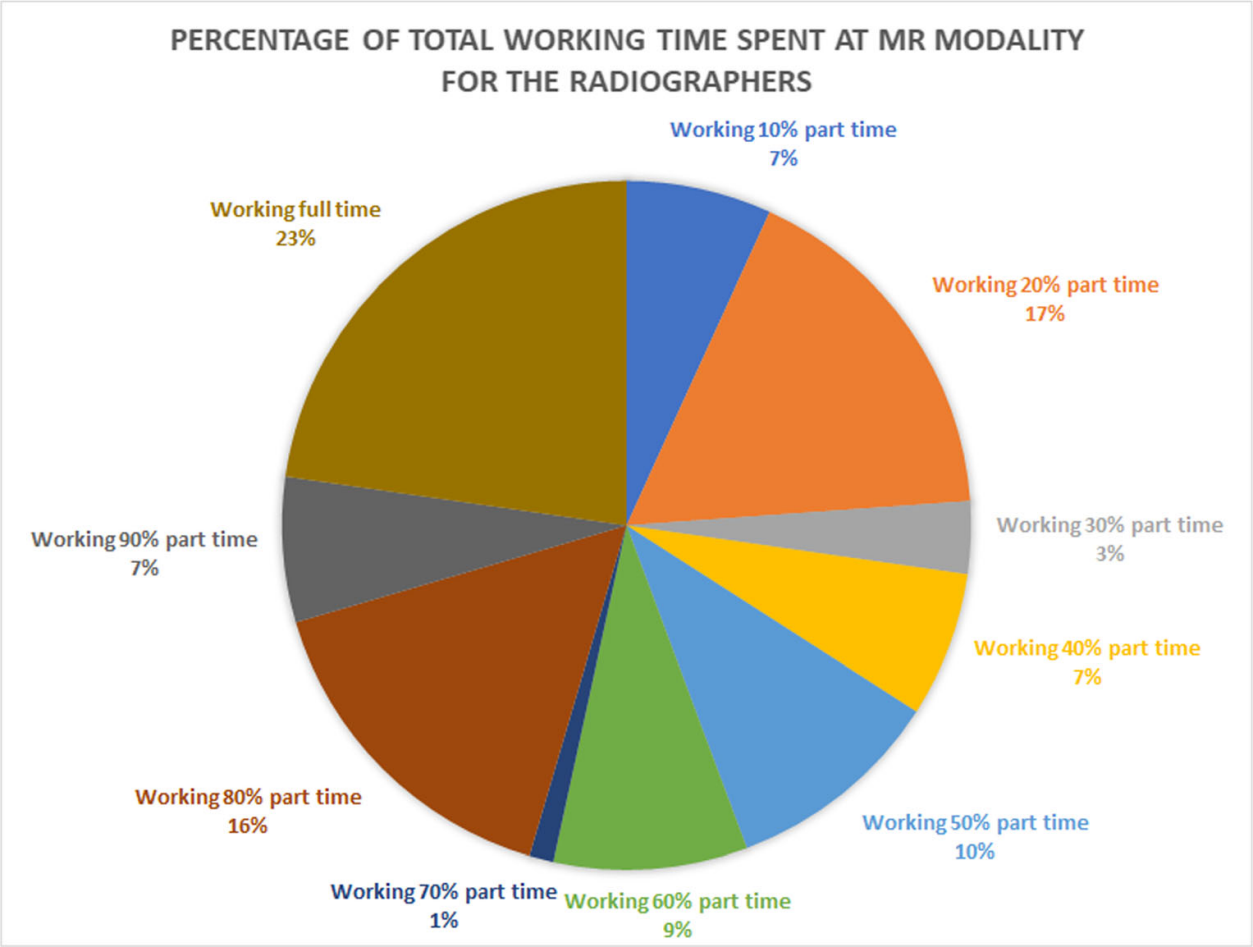
MR-specialised radiographers’ working time spent at an MR modality. Percentage of the 88 MR-specialised radiographers in 13 MR sites divided into groups according to the percentage of the total working time spent in their MR modalities

There were a total of 88 radiographers specialising in MR at the 13 sites, and 66% of them worked 50% of the time, or more with MR; see Fig. 5. Correlations between the ‘Fraction of work at MR’ and ‘Number of reported incidents’, as well as ‘MR knowledge’, were observed (Spearman’s rho 0.64 and 0.67, respectively). MR education was provided differently at the different sites, although informal education by peer-to-peer education at the site was common. Sixty-eight percent of the radiographers had attended university-level courses in MR, but some had instead attended vendor-provided courses on MR. Three of the radiographers had an academic master’s qualification, or above.

All responders reported that the radiologists at the site had the responsibility for risk-benefit analysis when deciding if patients with MR-conditional, or unsafe implants, would be examined or not. However, conventional content analysis showed that just one out of 13 responders (c. 92%) was confident that the radiologists had adequate knowledge of MR safety and the risks involved in operating a scanner. Spontaneous comments by radiographers such as ‘*Seldom enough knowledge*’, ‘*Not always enough knowledge*’ or ‘*They don’t always know what they are signing*’ were common. We chose not to follow up these comments with radiologists on each site, as that was beyond the aim of this study.

In seven MR sites (54%), the MR-specialised radiographer searched medical records for the presence of implants, and if unknown implants were found in this manner, an MR physicist was consulted, and a radiologist would then conduct a risk-benefit analysis. At the remaining sites (46%), the radiologist would search in the medical records, find MR conditions for the implant and conduct the risk-benefit analysis, very rarely consulting an MR physicist. Nine of the sites had MR physicists regularly involved in unknown, or complicated implant situations. Seven sites had assistant nurses helping the radiographer with the patient handling, and a few more considered engaging such assistant nurses.

Conventional content analysis showed also that all the responders wanted both more education in and collaboration on MR safety, and this was expressed, for example, as ‘*Migrate international recommendations into a Swedish context*’, ‘*Introduce national guidelines regarding implants*’, ‘*MR safety workshops*’, ‘*MR safety education in Swedish*’, ‘*Visit other MR units*’, ‘*Increase MR safety knowledge among radiologists*’ and ‘*The anaesthesia care teams need to learn more about MR safety*’, etc.

## Discussion

### Site-focused model

The questionnaire that was used in conjunction with the qualitative method content analysis provided credible results compared to previous studies. Moreover, new issues that were not specifically asked for emerged. The study should be repeated within a few years after educational efforts to investigate the impact of training intervention.

### Incidents and background factors

In this study, staff at 13 MR sites were interviewed using a semi-structured questionnaire about the level of MR safety and MR-related incidents at their site. The reporting rate of incidents was very low (38%), suggesting that about 100 incidents remained unreported. In a recent report, it was suggested that about 30% of incidents were unreported and undocumented. In that web survey, the number of documented incidents was around 1.5 incidents per scanner and year [16], compared to 0.4 found here. Perhaps the different methods used to ask about the number of incidents, i.e. an anonymous web survey versus an in-depth interview, might affect the actual level of reporting.

In the FDA incident management system (MAUDE), RF burns are the most common type of incident (59% of all incidents, [15]). In this study, about 7% of incidents were reported as RF burns. Attitudes such as ‘*Burns are to be expected once in a while*’, implying it was felt unnecessary to report such incidents, suggest that some of the missing reports could have been RF burns. The willingness to report incidents varied widely across the sites. One site routinely reported scanned patients that later were found to have coins or paper clips in their clothing. This reflects an accurate interpretation of Swedish law that defines incidents as ‘*Unwanted events that happened, or could have happened*’ [2]. Some sites did not report any unwanted events at all during the 5 years, something that may not be correct, and this could be an additional reason for the underreporting.

The most serious incidents were probably reported, but a few phenomena emerged that can be interpreted as reflecting an attitude toward MR safety. For example, it was said that RF burns are expected in MR, a few sites did not know the name of the incident reporting system, there were no reported incidents in 5 years in some sites and some sites were not able to search for MR incidents in the incident reporting system due to a lack of sufficient search features. To improve the safety culture in a clinical radiological context, actions such as education about the concept of ‘error prevention’ as well as methods for focusing attention to real incidents have previously been reported [21].

We did observe a negative correlation between ‘MR safety knowledge’ and the number of incidents, implying that more education is required. One contributing parameter could be that only 3% of the radiographers had an academic master’s degree or higher degree. This was also highlighted by the wish for more widely available safety training. If the MR operator’s knowledge about the motivation behind the questions in the screening forms was better, fewer patients would in our view likely be exposed to projectile accidents, or implant or bore-related RF burns. In Table 1, one site (#13) was recorded as 0% in ‘MR safety knowledge’, meaning that the lead MR radiographer did not think any of the MR operators would be knowledgeable enough to adjust commonly used safety-related scanning parameters such as SAR, SED or the effects of gradient performance. In-depth MR safety knowledge is important, and by improving knowledge about the influence of a number of scanning parameters, RF-related burns as well as implant malfunctions could perhaps be avoided. There are international organisations that provide various opportunities for education, including workshops and eLearning [13, 14], but those organisations were not mentioned in the interviews. Education in Swedish was asked for, but at present, there is no Swedish national body, or organisation, that can provide localised courses that would be as widely available as is requested. Clearly, improved means for mediating education need to be explored in future work.

There could be other obstacles to not reporting incidents apart from safety culture and lack of knowledge, such as complicated applications/software. Those obstacles did not occur in this material, but lack of time has been mentioned before [16].

Do staffing level and professional profile affect safety outcome? Here, the number of MR staff correlated with the number of incidents per year and per scanner, although only weakly so (Fig. 2, top left). We speculate that the division of work tasks might not be sufficiently well defined, so when several staff are working together, the MR safety is not fully covered, but when working alone, you are ‘the last man standing’. However, the more people who check the MR safety, the better it will be. International guidelines state that to be able to handle emergencies and to maintain MR safety at the site, there should be two staff members at a site with a single scanner [10, 14, 22] and at least three staff members in a dual scanner unit.

The MR personnel at some sites, for various reasons, cleaned the MR unit themselves. Other sites reported using external janitors, but they never left these workers unsupervised. The safety due to cleaning appeared to be high, and external janitors did not seem to affect MR safety in a negative way.

External personnel such as anaesthesia care teams typically contribute to an increased risk in the MR environment, as has been reported for example from Denmark [16]. These personnel are required in some examinations, but education is particularly important for those who work more seldom in the MR scanner environment, ‘seldom’ meaning that the MR safety routines are much more difficult to maintain, and to keep the anaesthesia personnel group small appears to be essential from an MR safety perspective, although this is a huge challenge in large hospitals.

MR-specialised radiographers have a very diverse background, and not surprisingly, MR knowledge varied substantially across the sites. The entire process of maintaining MR safety requires collaboration between different professions such as radiographers, MR physicists and radiologists. In this study, MR knowledge increased as a consequence of the availability of MR physicists at the site, and that appeared to decrease the risk for incidents. Neither experience nor knowledge about MR safety of the radiologists was investigated in this study. Nevertheless, almost all radiographers were concerned about their radiologists’ MR safety knowledge. The need to educate radiologists more extensively in MR safety has also been confirmed by Rajan et al [23].

### Outlook

There seems to be a continued need and a wish for additional and more easily available MR safety education for all professionals working with MR. The guarantor could be either a national agency or a non-profit professional association. The hospital’s management must also prioritise this matter. We intend to repeat this survey every 3 to 5 years, perhaps expanding it to also include MR safety knowledge and attitudes among radiologists and physicists.

### Limitations

The main limitation of this study is the relatively limited number of formally documented incidents, and this resulted in relatively weak, but significant, correlations. The questionnaire was developed and evaluated by an MR safety expert committee, although it was not properly validated. Some of the questions have previously been used in a national survey [17], and by supplementing these with additional open questions, the responders were able to openly discuss their thoughts and opinions. One additional limitation was that only a few respondents were interviewed at each site. These individuals could have forgotten some of the events, or perhaps they simply were not aware of all incidents. However, they received the questionnaire beforehand, which hopefully reduced that risk.

### Conclusions

This study showed that MR-related incidents are greatly underreported, and some of those incidents that actually do happen could potentially have catastrophic outcomes. To enhance the safety culture across all sites, more easily accessible education is needed. Broadening collaboration among radiographers, radiologists and MR physicists will also enhance the safety work.

## Supplementary Information

ESM 1 (DOCX 30 kb)

## Abbreviations

B0: Static magnetic field
B1: Time-varying electromagnetic fields
B1 + rms: Radio frequency transmission field
FDA: US Food and Drug Administration
MAUDE: Manufacturer and user facility device experience
MR: Magnetic resonance
PNS: Peripheral nerve stimulation
RF: Radio frequency
SED: Specific energy dose
T: Tesla

## Declarations

### Guarantor

The scientific guarantor of this publication is Peter Lundberg.

### Statistics and biometry

No complex statistical methods were necessary for this paper.

### Methodology

• cross-sectional study

• multicentre study
